# Supplementary material for: Prevalence and risk factors of birth asphyxia at Livingstone University teaching hospital
Source: PLOS Glob Public Health. 2025 Dec 16;5(12):e0004757. doi: 10.1371/journal.pgph.0004757 (PMC12707660; doi:10.1371/journal.pgph.0004757)
Supplement: Attachment — Submitted filename: General comment Plos.docx [file pgph.0004757.s003.docx]

**Prevalence and risk factors of birth asphyxia at Livingstone University Teaching Hospital**

**Abstract**: the method section needs revision for better clarity

**Introduction:** well written

**Methodology:** The methodology is comprehensive, clearly outlining the study design, setting, population, data collection, variables, and analysis approach. The use of a standardized tool (REDCap) and adherence to the STROBE checklist enhance the study's rigor. The definitions and statistical methods are appropriate for the research objectives. However, minor grammatical refinements and clarification on exclusion criteria (e.g., rephrasing “Mothers with incomplete records congenital anomalies...”) would improve clarity. Overall, the section effectively supports the study’s credibility and reproducibility.

**Result:** well written, But there are sample size inconsistency in table 1, justify or correct it.

After adjusting for confounders in multivariable models, these same four variables remained independently significant: eclampsia conferred a 19-fold increase in odds (AOR = 19.3; 95% CI: 3.0–123.0; p = 0.0017), very wide CI? Recheck.

Table 2, is it univariate or bivariate? Recheck.

**Discussion:** well done

**Conclusion:** well done

**References:** well done
