# Supplementary material for: Prevalence and risk factors of birth asphyxia at Livingstone University teaching hospital
Source: PLOS Glob Public Health. 2025 Dec 16;5(12):e0004757. doi: 10.1371/journal.pgph.0004757 (PMC12707660; doi:10.1371/journal.pgph.0004757)
Supplement: Attachment — Submitted filename: Responses to reviewer comments.docx [file pgph.0004757.s004.docx]

**Responses to Reviewers**

Reviewer #1:

1. Study design cannot be a retrospective cross-sectional. It can be either secondary data analysis of medical records or retrospective chart review.
   **Response: We thank the reviewer for this crucial clarification. We have revised the manuscript to describe the study design consistently as a 'secondary data analysis of medical records' throughout the abstract, methods, and other relevant sections.**
2. Data analysis plan is inadequate. The criteria for selection of variables for multivariable regression analysis are not described. The p-value cutoff must also be mentioned.
   **Response: We thank the reviewer for this important feedback. We have now revised the 'Data Analysis' subsection in the methodology to provide a detailed description of our variable selection process. All variables with a p-value < 0.2 in the bivariate analysis were considered for inclusion in the initial multivariable logistic regression model. A stepwise backward elimination approach was then used, retaining variables with a p-value < 0.05 in the final model. This p-value cutoff for significance is now explicitly stated.**
3. Replace univariable with Univariate.
   **Response: Thank you, we have changed this to bivariate throughout the manuscript, as this more accurately describes the analysis of the relationship between each independent variable and the outcome**.
4. If CIs are reported, the p-value is not necessarily required.
   **Response: Thank you for this suggestion. We agree and appreciate this. In order to also reach wider public who may not understand the significance of CI when placed alone, we have decided to retain the p-values for the odds ratios where the confidence intervals are reported. However, we are happy to remove them if the reviewer recommends so.**
5. There are AORs for all variables, a few are not even significant. Are they still in the model? The author needs to clearly describe the process and rationale for keeping variables in multivariable models.
   **Response: We appreciate this comment. The final multivariable model presented now only includes the variables that were statistically significant (p < 0.05). We have clarified this in the results section and in the caption for Table 2. The methodology section now explicitly details our model-building process (as described in point #2 above), which justifies the variables included in the final model.**
6. Improve flow by reducing repetitive phrasing and fixing grammatical errors.
   **Response: Thank you for checking grammatical errors. We have thoroughly proofread the entire manuscript to improve sentence flow, eliminate repetitive phrasing, and correct grammatical errors.**

**Reviewer #2**

Abstract: the method section needs revision for better clarity
**Response: We agree and thank the reviewer for this suggestion. We have revised the methods section of the abstract for better clarity and conciseness.**

Introduction: well written
**Response: Thank you.**

Methodology: The methodology is comprehensive, clearly outlining the study design, setting, population, data collection, variables, and analysis approach. The use of a standardized tool (REDCap) and adherence to the STROBE checklist enhance the study's rigor. The definitions and statistical methods are appropriate for the research objectives. However, minor grammatical refinements and clarification on exclusion criteria (e.g., rephrasing “Mothers with incomplete records congenital anomalies...”) would improve clarity. Overall, the section effectively supports the study’s credibility and reproducibility
**Response: We appreciate this feedback. We have carefully proofread the entire methodology section for grammatical precision. Furthermore, we have rephrased the exclusion criteria for absolute clarity to: "Neonates with incomplete medical records or those with major congenital anomalies unrelated to birth asphyxia were excluded."**

Result: well written, But there are sample size inconsistency in table 1, justify or correct it.
**Response: We thank the reviewer for identifying this. The varying sample sizes (n) for each variable in Table 1 are not an inconsistency but a common feature of retrospective data analysis, reflecting missing data in specific fields within the medical records (e.g., a field for 'anaemia' or 'eclampsia' was sometimes left blank). To prevent misinterpretation, we have added a note beneath the title of Table 1 to clarify this: "Note: The total N for each variable varies due to missing data in the medical records."**

After adjusting for confounders in multivariable models, these same four variables remained independently significant: eclampsia conferred a 19-fold increase in odds (AOR = 19.3; 95% CI: 3.0–123.0; p = 0.0017), very wide CI? Recheck.
**Response: The reviewer is correct to note the very wide confidence interval for eclampsia. We have rechecked the analysis, and the result is accurate. The wide CI (3.0–123.0) is a statistical reality due to the very low number of eclampsia cases in our cohort (n=9 total, with only 3 cases in the asphyxia group). While this leads to imprecision in estimating the exact magnitude of the effect, the strong statistical significance (p=0.0017) consistently indicates a very large and robust association. We have acknowledged this limitation explicitly in the discussion section.**

Table 2, is it univariate or bivariate? Recheck.
**Response: Thank you. We have revised this to the correct term, 'bivariate', in the table title and throughout the manuscript.**

Discussion: well done
**Response: Thank you.**
Conclusion: well done
**Response: Thank you.**
References: well done
**Response: Thank you.**
