# Supplementary material for: Prevalence and risk factors of birth asphyxia at Livingstone University teaching hospital
Source: PLOS Glob Public Health. 2025 Dec 16;5(12):e0004757. doi: 10.1371/journal.pgph.0004757 (PMC12707660; doi:10.1371/journal.pgph.0004757)
Supplement: Attachment — Submitted filename: Response to Editor and Reviewer Comments.docx [file pgph.0004757.s005.docx]

**Response to Editor Minor Comments**

**Define abnormal cardiotocography and define prolonged labour as it was associated with higher rates of fetal distress and fetal compomise in labour.**

**Differentiate abnotmal CTG and fetal distress. What is the difference?:**

Response to comment 1 and 2

We thank the editor for this important clarification. We have revised the 'Methods' section to include explicit definitions for both terms.

Abnormal cardiotocography (CTG) is now defined as a tracing that exhibits patterns classified as pathological or suspicious according to the FIGO 2015 guidelines.

Prolonged labour is defined based on established duration limits for the first and second stages.

Furthermore, we have clarified the distinction between an abnormal CTG and fetal distress. We now state that an abnormal CTG is a screening tool indicating concerning fetal heart rate patterns, while a diagnosis of fetal distress reflects a clinical judgment of confirmed fetal compromise, which is typically supported by the abnormal CTG in conjunction with other signs, such as meconium-stained amniotic fluid or fetal blood sampling results.

**Revise English language please. For instance:**

**Table1. Avoid using questions in the variable definitions. "Did mother suffer prolapsed umbilical cord" to be replaced with "umbilical cord prolapse",. etc**

Response to comment 3

We have thoroughly revised the manuscript for language clarity and readability. As specifically requested, Table 1 has been updated to use concise, declarative variable names (e.g., “Umbilical cord prolapse”, “Antepartum haemorrhage”).

**Discuss the issue that normally prolonges second stage is associated with lower umbilical pH and neonatal acidemia and provide references. There are studies showing Quantification of Posterior Risk Related to Intrapartum FIGO 2015 Criteria for Cardiotocography in the Second Stage of Labor and others assessing Hazard and cumulative incidence of umbilical cord metabolic acidemia at birth in fetuses experiencing the second stage of labor and pathologic intrapartum fetal heart rate requiring expedited delivery. Some of this studies show a correlation with timing in the second stage: the longer the second stage the higher the risk of neonatal acidemia. Compare this with their finding and discuss this difference.**

Response: This is an excellent point. We have now expanded the 'Discussion' section to directly address this established correlation. We acknowledge that several contemporary studies, including those referenced by the editor, demonstrate a clear time-dependent relationship between the duration of the second stage and the risk of neonatal acidemia, particularly in the context of pathological FHR patterns.

We discuss that our finding (where prolonged second stage was not an independent predictor of acidemia in the final model) may seem contradictory. We propose potential explanations for this discrepancy, such as the proactive management of labour in our cohort, where expedited delivery (via operative vaginal or cesarean delivery) may have been initiated upon recognition of a pathological CTG, thereby interrupting the pathway to acidemia. This intervention could attenuate the direct statistical association between time and outcome in our analysis. This added discussion provides a more nuanced interpretation of our results within the context of current literature.
